# SARS-CoV-2 seroprevalence and determinants for salivary seropositivity among pupils and school staff: a prospective cohort study

**DOI:** 10.1017/S0950268823000584

**Published:** 2023-04-24

**Authors:** Joanna Merckx, Milena Callies, Ines Kabouche, Isabelle Desombere, Els Duysburgh, Mathieu Roelants

**Affiliations:** 1 Department of Epidemiology, Biostatistics and Occupational Health, McGill University, Montreal, QC, Canada; 2 Department of Epidemiology and Public Health, Sciensano, Brussels, Belgium; 3 Department of Infectious Diseases in Humans, Immune Response, Sciensano, Brussels, Belgium; 4 Environment and Health, Department of Public Health and Primary Care, KU Leuven, Leuven, Belgium

**Keywords:** Cohort, determinants, oral samples, SARS-CoV-2, schools, serology

## Abstract

Representative school data on SARS-CoV-2 past-infection are scarce, and differences between pupils and staff remain ambiguous. We performed a nation-wide prospective seroprevalence study among pupils and staff over time and in relation to determinants of infection using Poisson regression and generalised estimating equations. A cluster random sample was selected with allocation by region and sociodemographic (SES) background. Surveys and saliva samples were collected in December 2020, March, and June 2021, and also in October and December 2021 for primary pupils. We recruited 885 primary and 569 secondary pupils and 799 staff in 84 schools. Cumulative seroprevalence (95% CI) among primary pupils increased from 11.0% (7.6; 15.9) at baseline to 60.4% (53.4; 68.3) in December 2021. Group estimates were similar at baseline; however, in June they were significantly higher among primary staff (38.9% (32.5; 46.4)) compared to pupils and secondary staff (24.2% (20.3; 28.8)). Infections were asymptomatic in 48–56% of pupils and 28% of staff. Seropositivity was associated with individual SES in pupils, and with school level, school SES and language network in staff in June. Associations with behavioural characteristics were inconsistent. Seroconversion rates increased two- to four-fold after self-reported high-risk contacts, especially with adults. Seroprevalence studies using non-invasive sampling can inform public health management.

## Introduction

Uncertainty regarding the transmission of SARS-CoV-2 led to the implementation of non-pharmaceutical interventions and school closures. By fall 2020, most European countries and the UK [1] reinitiated in-person learning. Both closures and infection prevention and control (IPC) measures come with a cost to the development of children [2], but remained in place without systematic investigation of their evidence base. In Belgium, schools were closed in March 2020 and partially reopened in April 2020. The 2020–2021 academic year started with in-person learning for all pupils, subject to masking for staff and secondary school pupils, physical distance between pupils and staff, enforced hygiene measurements, and investment in school ventilation while the second pandemic wave surged. Boey et al. compared the seroprevalence in pupils from two Belgian regions in October 2020 and found that infection rates depended on the community incidence [3]. Travel, contact with a confirmed case, and a parent who is a healthcare worker (HCW) were identified as risk factors. SARS-CoV-2 vaccination started on 5 January in Belgium. Between January and April 2021, priority groups were vaccinated. These included staff and residents of nursing homes, HCWs, residents of long-term care facilities, ≥65-year-olds, persons with comorbidities, and pregnant women. The campaign was extended in May 2021 to certain professions, including school staff in some regions, and in June 2021 to the general population of 18 years and older. Vaccination became available in August 2021 for the population between ages 12 and 17 and was recommended for all children below 18 by the end of December 2021 [4, 5].

Representative national studies investigating seroprevalence in pupils and school staff and assessing past infection rates [6] are scarce [7]. Estimates from leftover laboratory samples [8] in the paediatric population lack generalizability and do not provide the necessary context to assess determinants of infection. The impact of COVID-19 waves and the role of determinants in a representative sample of school pupils and staff remained unknown, and to date, it is unclear how infection rates in primary or secondary school children relate to those observed in school staff or those reported in surveillance programmes.

We assessed the prevalence of anti-SARS-CoV-2 antibodies at five test periods in a representative sample of primary and secondary pupils and staff in Belgium between December 2020 and 2021. We compared these estimates with the COVID-19 community incidence and assessed the effect of sociodemographic (SES) and time-variable behavioural determinants and occurrence of symptoms on seropositivity.

## Methods

### Study design and participants

A two-stage cluster random sample of participants was prospectively followed from enrolment in December 2020 or September 2021 (study extension in primary school pupils) until June or December 2021. SARS-CoV-2 seroprevalence was estimated using saliva samples collected in December/January (T1), March (T2), and May/June 2021 (T3), and in pupils who participated in the study extension also in October (T4) and December 2021 (T5). Pupils and staff were recruited in the same schools with proportional allocation by province and SES background using publicly available indicators [9]. In the first stage, 41 clusters were selected and assigned an SES quantile, in which one primary and one secondary school were randomly selected from a list of all schools that provide general education. In the second stage, a convenience sample of 20 pupils and 10 staff were recruited per school. Pupils were mainly recruited in the third year of primary school (ages 7–9) and first 2 years of secondary school (ages 13–14), and staff were in contact with these pupils. A sample size of 820 pupils and 410 staff in primary and secondary schools was calculated based on an estimated seroprevalence of 6% and standard error (SE) of 2.3% in pupils and 10% (SE 3%) in staff and a design effect of two. Data from staff were censored from the time of vaccination or from March when the vaccination status in June 2021 was unknown.

### Data collection

The presence of anti-SARS-CoV-2 antibodies was determined in self-collected saliva samples using oral swabs (Oracol; Malvern Medical Developments, UK) under the supervision of a trained nurse at school. Samples were stored at 2–8°C upon collection and during transport to the laboratory. Participants or their parents/caretakers were invited to complete an online questionnaire (LimeSurvey) on SES characteristics and existing conditions at baseline and on behavioural characteristics (e.g. extracurricular activities, public transport, travel), contact with confirmed cases, COVID-19 episodes and symptoms, IPC measures (staff only) at each test period. The surveys were piloted and available in French and Dutch. The cumulative incidence of reported cases of SARS-CoV-2 in the school district 2 weeks prior to the test period served as a measure of community exposure [10].

### Laboratory analyses

Biological samples were analysed at the Immunology Laboratories of Sciensano using SARS-CoV-2 IgG ELISA (Wantai Bio-Pharm; cat n° WS-1396) customised for saliva using an in-house protocol. The assay measures anti-RBD (Receptor Binding Domain) IgG in centrifuged and diluted crevicular fluid. A specificity-optimised cut-off value >1.5 signal-to-noise ratio was used for seropositivity. Specificity and 95% confidence intervals (95% CI) of the assay were estimated (96.7% (90.8%–99.1%) and 96.5% (91.4%–98.6%)) at a sensitivity of 95.1% (86.5%–98.7) and 80.0% (58.4–91.9%) for adults and children, respectively. Assaying and test validation were performed using a pilot study in children [3], an adult HCW study [11], and an outbreak investigation (unpublished data; children and adults) and has been used in a population seroprevalence study that used oral sample collection through the mail [12].

### Data analysis

Point and cumulative seroprevalence and corresponding 95% CI were estimated at each of the five test periods using generalised estimation equations (GEEs) for intercept-only Poisson regression, with a log link function, exchangeable correlation structure (compound symmetry), and school as the clustering variable. The association of SES and seropositivity at baseline and in June 2021, as well as group differences (e.g. pupils versus staff, primary versus secondary schools), were estimated with analogous GEE Poisson (multiple) regression models. Results are expressed as risk differences (RD) or risk ratios (RR) by using an identity or log link function, respectively, and as partially adjusted RR (aRR), including school level (primary or secondary), school SES tertile, cumulative incidence in the school district 2 weeks prior to data collection as a measure of community exposure, and language network as a proxy for the different geographic regions of Belgium. Estimates for pupils were additionally adjusted for a vulnerable situation at home (defined as the presence of one or more of the following characteristics reported in the baseline survey at inclusion: lower education of mother or father, unemployed mother or father, household monthly budget <1500 EUR, financial situation reported as being difficult, language at home does not include Dutch, French, or German, as reported from the individual survey data), and those of the staff were adjusted for function (teaching or not), sex, and age. Potential determinants and confounders were identified using a directed acyclic graph based on the literature (Supplementary Figure S1). Determinants of seroconversion between two consecutive test periods were analysed with GEE Poisson regression of the cumulative serostatus, the determinant of interest, and relevant background variables at both time points using subject ID as the clustering variable. The impact of the determinant on seroconversion during the interval was derived from an interaction term with time and expressed as aRR with 95% CI. Case under-ascertainment was calculated at specific time points as the ratio of the cumulative seroprevalence to the self-reported PCR-confirmed infections or the national cumulative incidence of confirmed acute cases in comparable age groups (respectively 6–11, 12–14, and 18–64-year-olds).

Data were analysed with R version 4.0 (R Foundation for Statistical Computing, Vienna, Austria, 2020). The article was prepared using ROSES-S [13] and STROBE reporting guidelines [14].

### Funding and ethics

The study was approved by the Medical Ethics Committee of the University Hospital Ghent (reference B6702020000744 – BC-08564), funded by the Belgian government through Sciensano, and registered at ClinicalTrails.gov (NCT04613817) [15]. Written informed consent was obtained from staff and parents/legal guardians of the pupils before enrolment along with written informed assent from pupils.

## Results

### Participants’ characteristics

**Figure 1.**
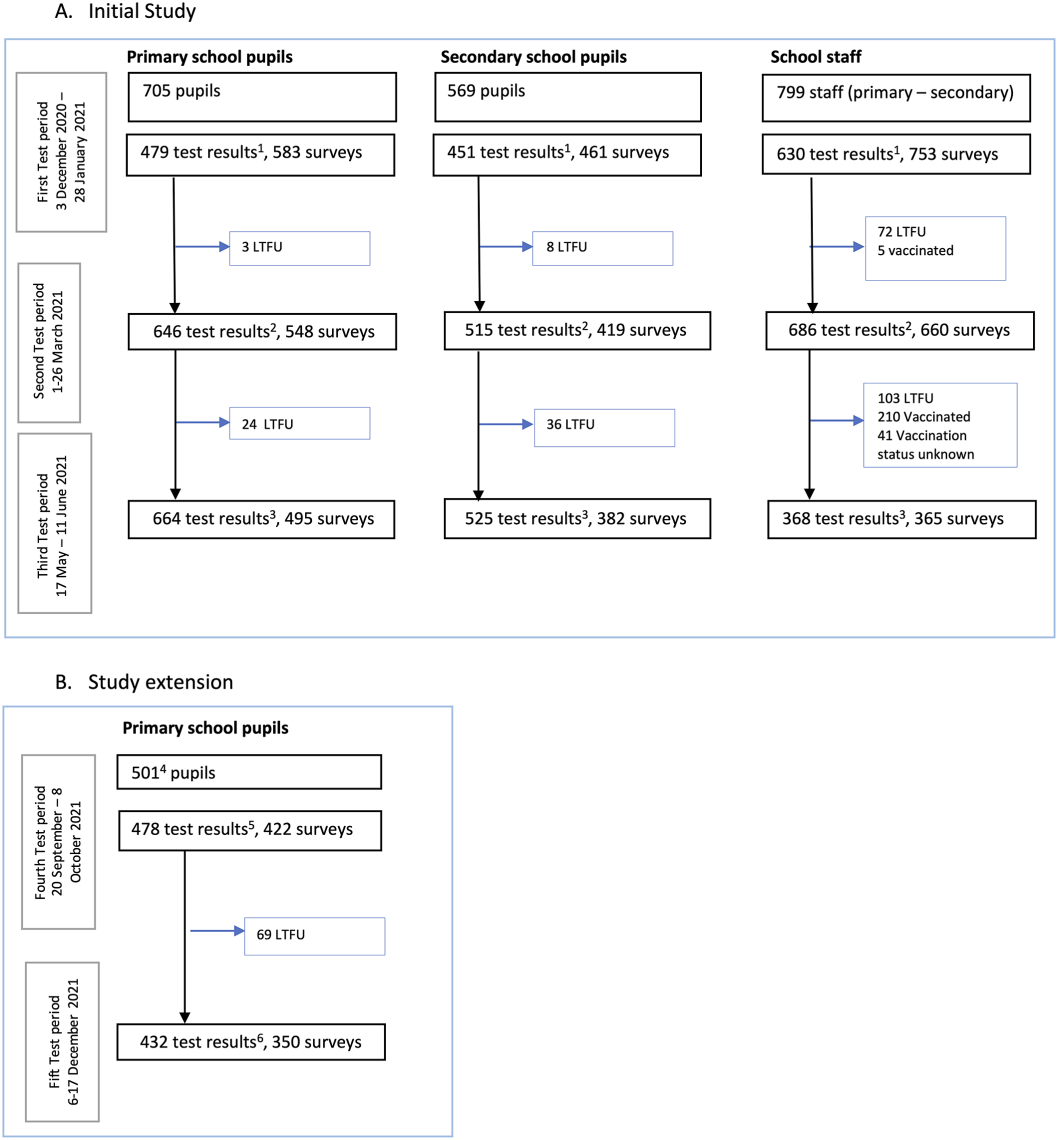
Flowchart of included participants by age group and test period. ^1^Excluding 503 out of 2063 (24%) samples with insufficient volume for reliable determination of antibodies at T1 in all age groups combined. ^2^Excluding 37 (2%) samples with insufficient volume. ^3^Excluding 25 (1%) samples with insufficient volume. ^4^Including 321 pupils from the initial study and 180 newly recruited pupils. ^5^Excluding six (1%) samples with insufficient volume. ^6^Excluding two (0.5%) samples with insufficient volume. LTFU, lost to follow-up; no data available after this period.

Eight hundred and eighty-five primary and 569 secondary school children and 799 staff from 44 primary and 40 secondary schools (Supplementary Figures S2 and S3) were enrolled in the study, but the number of samples and surveys differed by test period (Figure 1). The volume of 503 out of 2,063 (24%) collected samples at baseline was insufficient (<100 μl) for a reliable determination of antibodies. Additional training and quality control reduced this number to 2% or less at the next test periods (Figure 1). Non-participation or exclusion from the test periods was unrelated to seropositivity (data not shown). Out of 705 primary school pupils enrolled in December 2020, 321 participated in the study extension, and another 180 were enrolled in September 2021.

SES and household characteristics are presented in Table 1. Primary school pupils had a median age of 9 years and secondary school pupils of 14 years. About one-quarter of the pupils were considered vulnerable. Baseline characteristics of newly enrolled pupils were overall comparable to the primary study except for sex, and less participation of schools belonging to lower SES tertiles and to the French-language network (Supplementary Table S1). From October 2021 onwards, more than 90% of pupils lived in a family where all adults were vaccinated against SARS-CoV-2. The median age of staff was 41 years, and most were female. A chronic health condition was self-reported for 3% of primary and 8% of secondary pupils and by 10% of staff. Five staff members were vaccinated between January and March 2021 and another 210 between March and May 2021; 41 were excluded in June because their vaccination status was unknown.

**Table 1.** SES and household characteristics of participants

	Pupils *N* (%)		
	Primary school	Secondary school	Staff *N* (%)
Total number	885 (100%)	569 (100%)	799 (100%)
Sex (F/M, %F)	420/465 (47%)	270/299 (47%)	587/212 (73%)
Age (years), median (range)^a^	9 (6–12)	14 (13–16)	41 (21–66)
Language network			
Dutch/French	510/375 (58%/42%)	336/233 (59%/41%)	431/368 (54%/46%)
Region			
Brussels	120 (14%)	46 (8%)	94 (12%)
Flanders	488 (55%)	336 (59%)	421 (53%)
Wallonia	277 (31%)	187 (33%)	284 (35%)
School SES			
Low	294 (33%)	165 (29%)	273 (34%)
Intermediate	331 (37%)	178 (31%)	287 (36%)
High	260 (29%)	226 (40%)	239 (30%)
Staff function			*N* = 761
Teaching staff	–	–	616 (81%)
Support staff			145 (19%)
Origin^b^	*N* = 723	*N* = 456	
Belgium	499 (69%)	353 (77%)	–
Europe	119 (16%)	69 (15%)	
Out of Europe	105 (15%)	34 (7%)	
Language spoken at home	*N* = 713	*N* = 447	–
Including Dutch, French, or German	693 (97%)	439 (98%)	
Others, not including Dutch, French, or German	20 (3%)	8 (2%)	
Maternal/paternal education	*N* = 706/*N* = 699	*N* = 442/*N* = 437	–
Lower	38 (5%)/50 (7%)	19 (4%)/33 (8%)	
Secondary	194 (27%)/288 (41%)	142 (32%)/194 (44%)	
Higher	474 (67%)/361 (52%)	281 (64%)/210 (48%)	
Maternal/paternal occupation	*N* = 668/*N* = 660	*N* = 420/*N* = 402	–
Mother employed/father employed	584 (87%)/608 (92%)	378 (90%)/380 (95%)	
NACE group COVID-19 exposure risk^c^	*N* = 588	*N* = 369	–
Low	284 (48%)	176 (48%)	
High	304 (52%)	193 (52%)	
Monthly family household budget	*N* = 546	*N* = 315	–
<3000 Euro	173 (32%)	90 (29%)	
3000–5000 Euro	246 (45%)	151 (48%)	
>5000 Euro	127 (23%)	74 (23%)	
Financial ease	*N* = 711	*N* = 449	*N* = 741
Difficult	81 (11%)	55 (12%)	41 (6%)
Average	329 (46%)	222 (50%)	349 (47%)
Easy	301 (42%)	172 (38%)	351 (47%)
SES vulnerable pupil^d^	*N* = 731	*N* = 461	–
Any vulnerable characteristic	209 (29%)	120 (26%)	
Family composition^e^	*N* = 714	*N* = 448	*N* = 734
Core family	566 (79%)	322 (72%)	583 (79%)
Second residence	86 (12%)	79 (18%)	–
Family size	*N* = 712	*N* = 447	*N* = 696
Small (1–2 individuals)	34 (5%)	19 (4%)	264 (38%)
Medium (3–5 individuals)	582 (82%)	363 (81%)	401 (58%)
Large (more than 5)	96 (13%)	65 (15%)	31 (4%)
Children with shared bedroom	*N* = 714	*N* = 448	–
Shared room with other child, adult, or both	267 (37%)	86 (19%)	
Self-reported comorbidities participants^f^	*N* = 722	*N* = 455	*N* = 742
One comorbidity	16 (2%)	34 (7%)	77 (10%)
Two or more comorbidities	4 (1%)	1 (<1%)	16 (2%)
Smoking	–	–	*N* = 612
Current smoker			79 (13%)

a Missing age: 6 (<1%) primary school pupils; 41 (5%) staff.

b A child is considered of ‘Belgian origin’ when both parents and all grandparents were born in Belgium, ‘European origin’ when one or more parents or grandparents were born in a European country or the USA, Canada, or Australia/New Zealand, or ‘out of Europe’ when one or more parents or grandparents were born in a non-European country, not including the USA, Canada, or Australia/New Zealand.

c NACE group COVID-19 exposure risk: adapted from the classification by Konstantinos Pouliakas and Jiri Branka, ‘EU Jobs at Highest Risk of Covid-19 Social Distancing: Will the Pandemic Exacerbate Labour Market Divide?’, IZA Discussion Paper No. 13281, available at SSRN: https://ssrn.com/abstract=3608530 or https://doi.org/10.2139/ssrn.3608530, based on the European Skills and Jobs Survey (ESJS) with weighted scores based on COVID-19 exposure risk. Education and health services (NACE P,Q) were included in the high-risk group, being direct under the cut-off of 1 standard deviation from the mean of exposure risk. Parenthetically, parents reported they had an occupation in education and the healthcare sector with an *N* = 94 (16%) and *N* = 147 (25%), and *N* = 42 (11%) and *N* = 86 (23%) of participants from primary and secondary schools, respectively.

d A vulnerable pupil was defined as presence of one or more of the following characteristics: lower education of mother or father, unemployed mother or father, household monthly budget <1500EU, financial ease: difficult, language at home does not include Dutch, French, or German.

e Core family was defined as pupil living with both parents in the same household. Single-parent families account for 86 (12%) and 68 (15%) of the pupils’ family compositions for primary and secondary schools, respectively. New composite families and other family compositions: foster family: two primary, two secondary school pupils; living with grandparent: one primary school pupil; institution: one primary school pupil. Secondary residence: pupil living on a regular basis with other parent, with other adult or in an institution.

f Specific comorbidities of primary school children: eight respiratory, one cardiovascular, four immunodeficiency, one renal, one genetic, three gastroenteric, one neurological, six other; secondary school pupils: seven diabetes, 13 respiratory disease, one cardiovascular, one immunodeficiency, three renal, one gastroenteric, four neurological, six other; staff: 13 diabetes, 20 respiratory, 29 cardiovascular, seven immunodeficiency, three renal, three genetic, three cancer, nine gastroenteric, five neurological, 21 other.

### Seroprevalence

**Figure 2.**
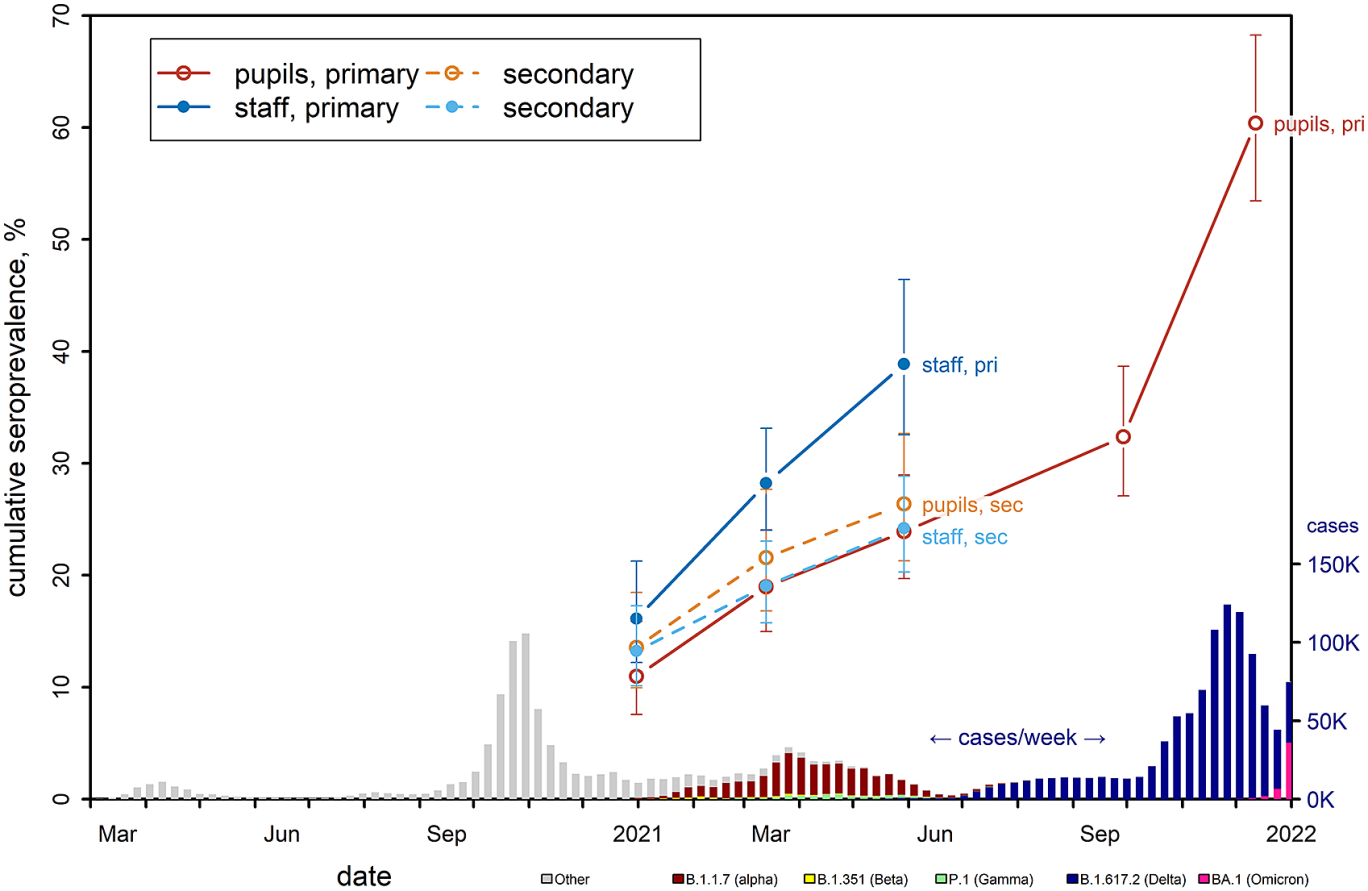
Cumulative seroprevalence (95% CI) in pupils (primary and secondary) and staff (primary and secondary) at each test period. Bars at the bottom show the weekly number of new cases of SARS-CoV-2 in the community with colours indicating the fraction attributable to specific variants of concern. ^1^Weekly new cases and distribution of variants from the Sciensano dashboard https://epistat.wiv-isp.be/covid/. *K* = *x*1000.

The prevalence of antibodies in December 2020 ranged from 11.0% (95% CI 7.6; 15.9) among primary school pupils to 16.1% (12.2; 21.3) among primary school staff, but differences were not statistically significant (Figure 2 and Supplementary Table S2). Sequentially, the cumulative incidence increased almost linearly in all age groups until June 2021, but the slope was steeper in primary school staff, reaching a prevalence of 38.9% (32.5; 46.4), compared to 23.9% (19.7; 29.0) among primary school pupils (RD 5.3% (95% CI 0.0; 10.6) in December; 14.3% (95% CI 6.6;22.1) in June). Differences were small and not statistically different between pupils from primary and secondary schools (Table 2 and Supplementary Table S3). Staff from primary schools were found to be more often seropositive compared to staff from secondary schools in March (RR 1.48; RD 9.2% (95% CI 3.4; 15) and June (1.61; 14.5% (6.3; 22.7)). No statistically significant differences were observed between secondary school pupils and their staff. In the study extension, cumulative seroprevalence continued to increase approximately linearly over the summer of 2021 (RD +7.3 percentage points (95% CI 3.9; 10.9) from June to October 2021) but accelerated between October and December 2021 (RD +27.7% (95% CI 23.5; 31.9)) to 60.4% (95% CI 53.4; 68.3%). Throughout the study, the intraclass correlation ranged from 0.01 to 0.03 in staff and from 0.04 to 0.1 in pupils.

**Table 2.** Relative risks (RR) for past infection (cumulative seroprevalence) in primary schools versus secondary schools and staff versus pupils point estimate, corrected for clustering

		Secondary vs. primary		Staff vs. pupils	
	2021	Pupils	Staff	Primary	Secondary
Test period 1	DEC–JAN	1.27 (0.78; 2.06)	0.82 (0.56; 1.21)	1.48 (0.99; 2.22)	1.02 (0.68; 1.54)
Test period 2	MAR	1.14 (0.81; 1.60)	0.67 (0.53; 0.87)	1.46 (1.15; 1.87)	0.89 (0.68; 1.17)
Test period 3	MAY–JUN	1.10 (0.83; 1.47)	0.62 (0.48; 0.81)	1.60 (1.26; 2.03)	0.98 (0.74; 1.31)

Test periods: DEC–JAN: 2020-12-03 to 2021-01-28; MAR: 2021-03-01 to 2021-03-26; MAY–JUN: 2021-05-17 to 2021-06-11; SEP–OCT: 2021-09-20 to 2021-10-08; DEC: 2021-12-07 to 2021-12-17.

### Symptomatology

Depending on the test period, between 35% and 61% of participants reported one or more COVID-19-related symptom. None of the participants reported being hospitalised for COVID-19 during the study. The sensitivity of COVID-19-related symptoms in previously seronegative participants ranged from 27% to 81% (Supplementary Figure S4). The majority of those who tested positive reported two or more symptoms. Loss of smell or taste was reported by 6% of pupils and 21% of staff who seroconverted. More importantly, 48% and 56% of primary and secondary school pupils and 28% of staff who tested positive did not report any symptom (Table 3).

**Table 3.** Asymptomatic cases in pupils and staff who tested positive for the first time in each test period: N asymptomatic/Total number of new positive cases with sufficient information (questionnaire on symptoms) and percentage

Age group	T1	T2	T3	T4	T5	overall
Pupils in primary school	17/45	19/26	21/45	14/24	41/93	112/233
	(38%)	(73%)	(58%)	(58%)	(44%)	(48%)
Pupils in secondary school	20/42	17/24	6/11	–	–	43/77
	(48%)	(71%)	(55%)			(56%)
Staff	16/83	14/41	11/22	–	_	41/146
	(19%)	(34%)	(50%)			(28%)

T1: December 2020/January 2021; T2: March 2021; T3: May/June 2021; T4: Sept/October 2021; T5: December 2021.

### PCR positivity and comparison with community cases

**Figure 3.**
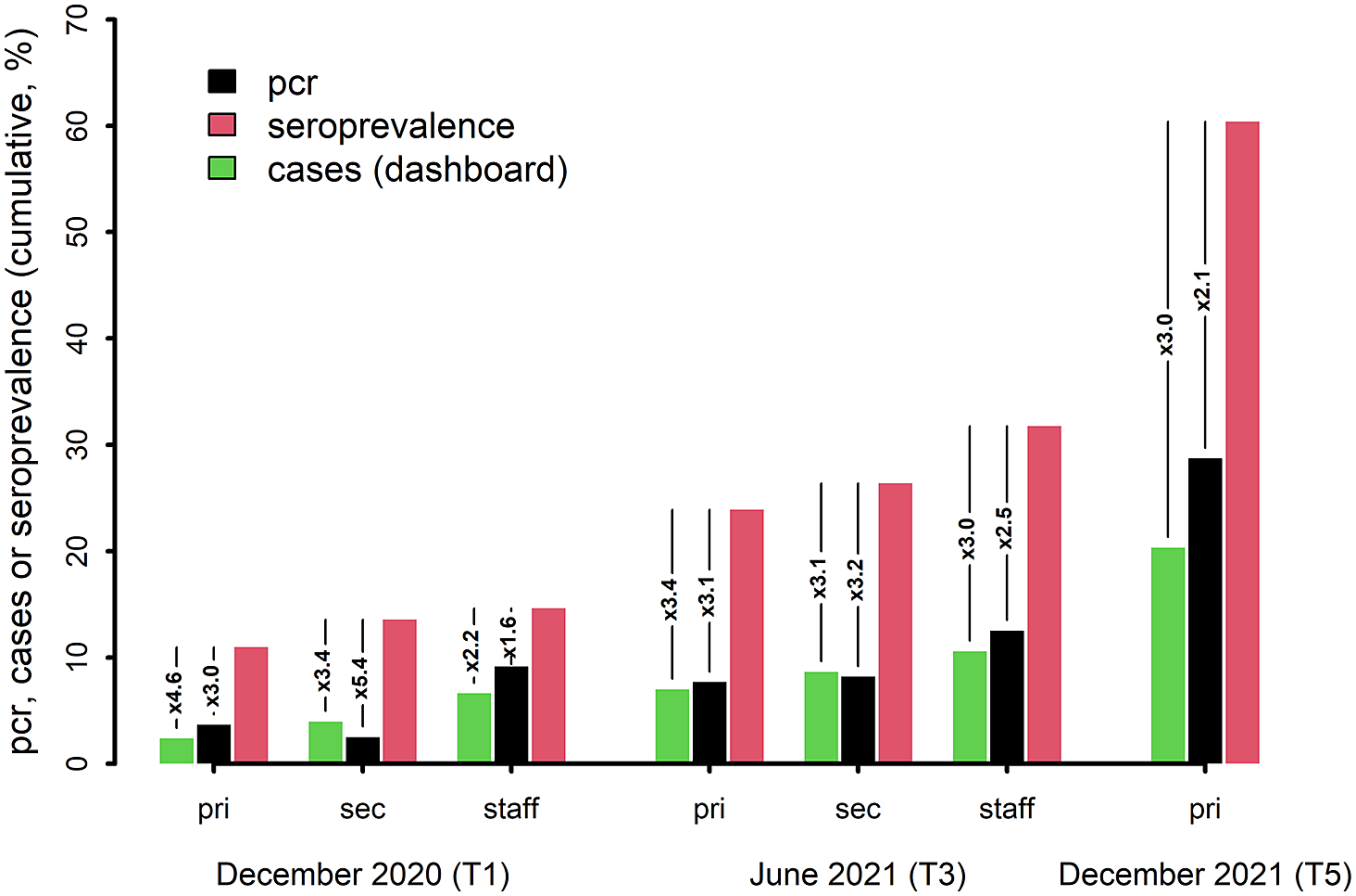
Under-ascertainment of cumulative past infection when comparing age-specific community-reported cases of acute infection (dashboard) or a previously self-reported positive PCR test in the study population with the cumulative seroprevalence at T1, T3, and T5. *x*: Both community cases (dashboard) and self-reported PCR tests severely underestimate infection rate estimated from the seroprevalence. ‘*x*’ indicates the factor to which the dashboard or PCR-based estimates should be multiplied (under-ascertainment). Data presented by age group: pri: primary school children or community cases in 6–12-year-old children; sec: secondary school pupils or community cases in 12–15-year-old adolescents, and staff or community cases in 18–65-year-old adults. T1: December 2020/January 2021; T2: March 2021; T3: May/June 2021; T4: Sept/October 2021; T5: December 2021.

By June 2021, 57 (7.7%) primary, 32 (8.2%) secondary school pupils, and 96 (12.5%) staff had reported a laboratory-confirmed acute SARS-CoV-2 infection (Figure 3), while the number of positive antibody tests was 2.1–3.1 times higher in pupils and 1.6–2.5 times higher in staff. Analogously, we observed a 2.0–4.6 times higher seroprevalence compared to the corresponding age-specific cumulative community prevalence (Figure 3).

### SES, health, and school-related determinants

**Figure 4.**
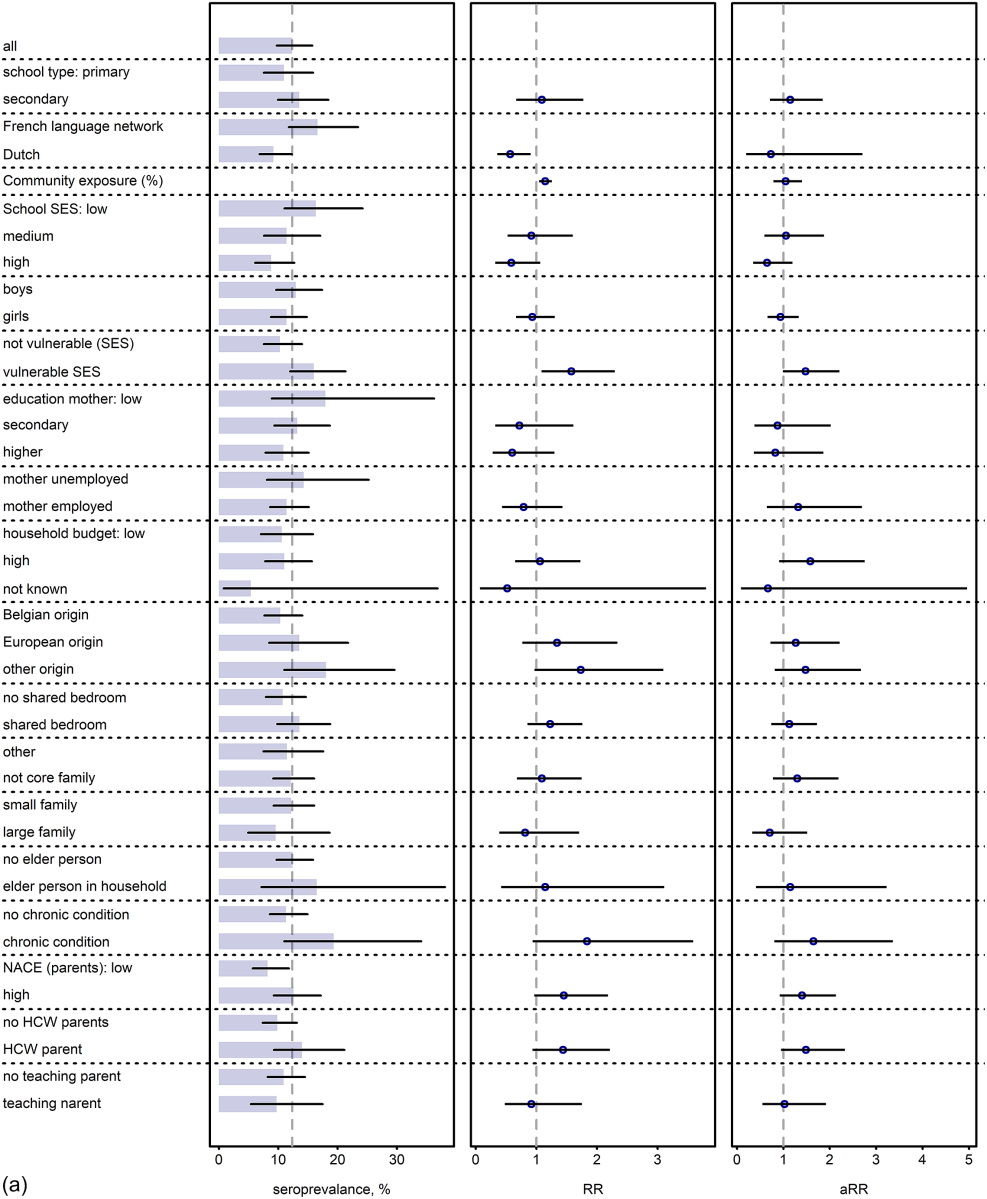
(a,b) SES determinant analysis reporting marginal seroprevalence, unadjusted risk ratio (RR) and partially adjusted^1^ risk ratio (aRR) in pupils (a) and staff (b) at test period 1 (baseline) with 95% confidence intervals. (a) ^1^Adjusted for school type (primary versus secondary school), language network (Flemish versus French), district-level cumulative community exposure, SES school, sex (female versus male), and being a vulnerable pupil defined as presence of one or more of the following characteristics: lower education of mother or father, unemployed mother or father, household monthly budget <1500EUR, financial situation reported as being difficult, language at home does not include Dutch, French, or German. All exposures were investigated in separate models; only the primary exposure is reported (aRR). HCW, healthcare worker; SES, socioeconomic status. (b) ^1^Adjusted for school type (primary versus secondary school), language network (Flemish versus French), district-level cumulative community exposure, SES school, sex (female versus male), staff function (teaching, non-teaching), presence of comorbidity, and age (>50 years). All exposures were investigated in separate models; only the primary exposure is reported (aRR). HCW, healthcare worker; SES, socioeconomic status.

Seroprevalence by determinants at baseline is presented in Figure 4 (corresponding numbers in Supplementary Table S4). In pupils, French-language network schools, community exposure, and being a vulnerable pupil (RR 1.58, 95% CI 1.09; 2.30) were associated with a higher risk of seropositivity in the unadjusted models at baseline; however, there was insufficient evidence of a statistically significant difference in the adjusted model (vulnerable pupil aRR 1.47 (95% CI 0.98; 2.19)). Although disadvantaged groups with respect to school and individual SES characteristics, large families, elderly household members, and occupational risk of parents were usually associated with a higher seroprevalence, none of these were statistically significant. In staff, at baseline, none of the determinants were associated with a significant different risk. However, analysis in June 2021 showed a statistically significant lower risk to test positive in schools of the Dutch-language network and from the highest and middle SES tertile (aRR 0.65; 95% CI 0.46; 0.91 and 0.73; 0.55; 0.97) (Supplementary Figure S5 and Supplementary Table S5).

**Figure:**
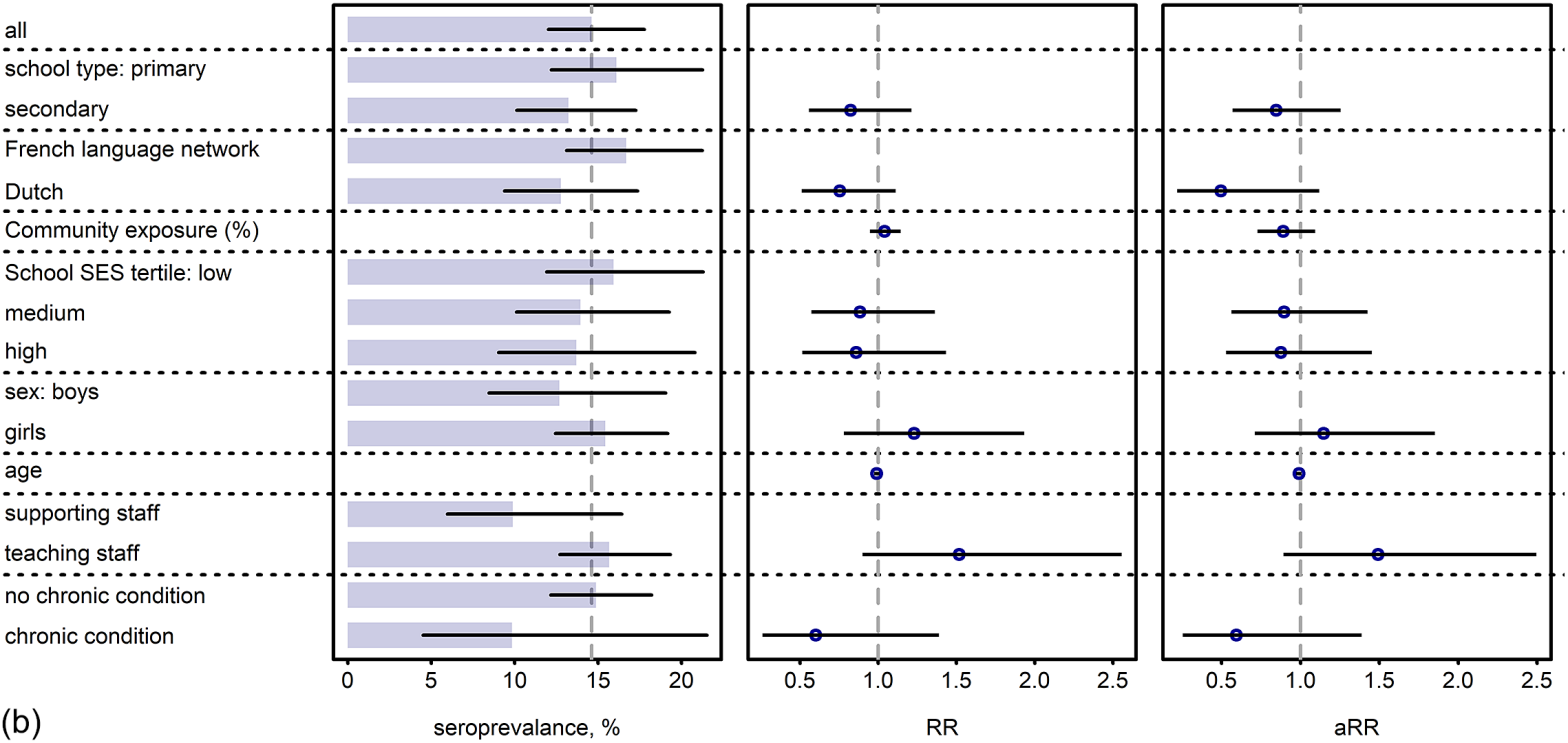

**Figure 5.**
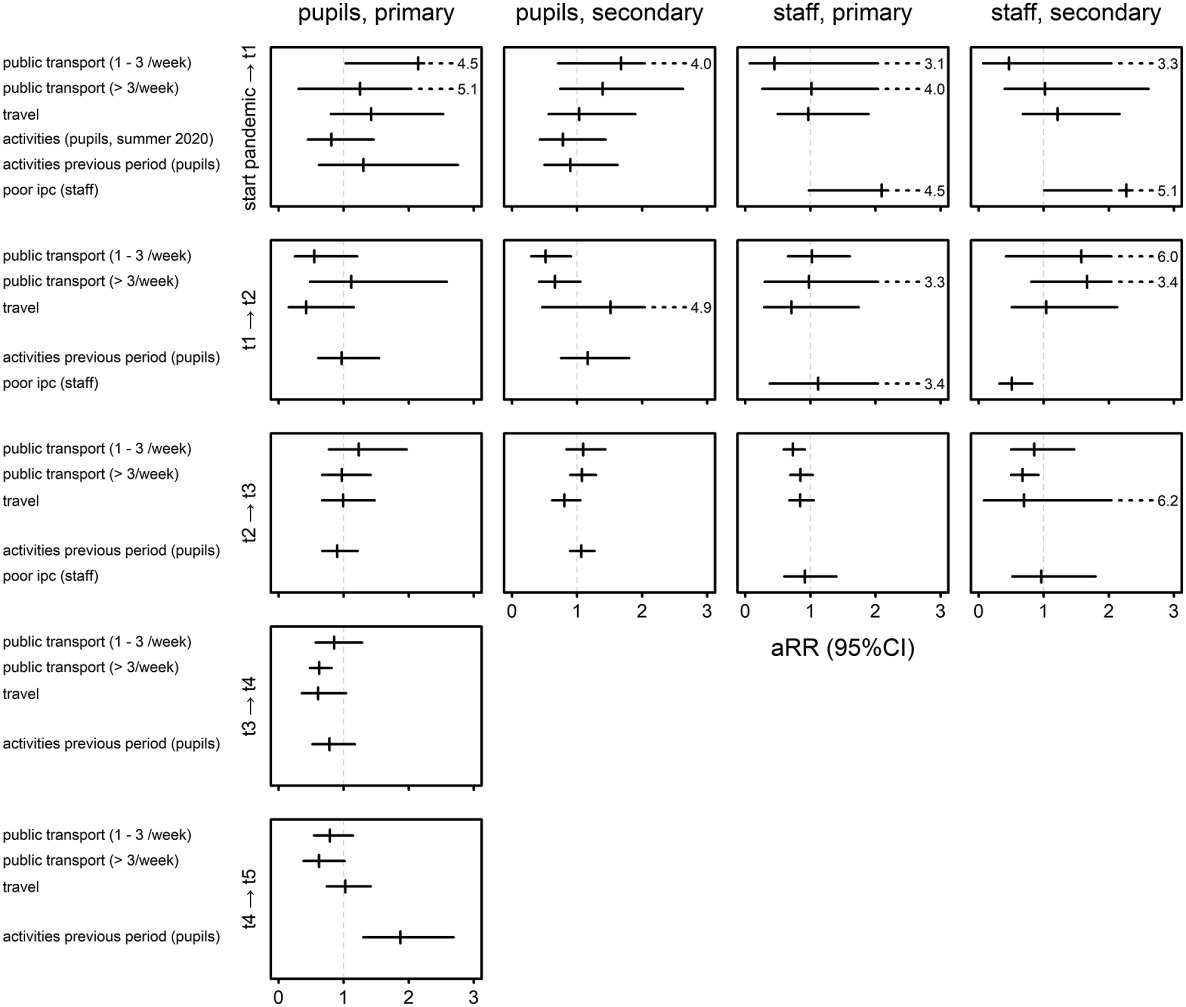
Associations between behaviour and seroconversion at baseline (i.e. since the start of the pandemic) and between consecutive test periods (T1 to T5) and by type of school (primary, secondary) and subject (pupil, staff) in previously seronegative participants, expressed as adjusted relative risks (aRR) with 95% confidence intervals. Public transport 1–3/week and >3/week compared to less than once a week; travel abroad by the participant or a family member versus no travel abroad; extracurricular activities (camp or other >3 hours/week) versus no extracurricular activities. At baseline, activities were questioned separately for the summer of 2020 and the school year (September 2020 and onwards); poor IPC: poor infection prevention and control implementation by the surveyed staff (<50% of the maximum score on a five-point scale for implementation and adherence of seven IPC measures). Models for pupils are adjusted for school type (primary versus secondary school), language network (Flemish versus French), district-level cumulative community exposure, SES school, sex (female versus male), and being ‘vulnerable’ defined as presence of one or more of the following characteristics: lower education of mother or father, unemployed mother or father, household monthly budget <1500EU, financial ease: difficult, language at home does not include Dutch, French, or German. Exposures were investigated separately; only primary exposures are reported (aRR). Models for staff are adjusted for school type (primary versus secondary school), language network (Flemish versus French), district-level cumulative community exposure, SES school, sex (female versus male), staff function (teaching, non-teaching), presence of comorbidity, and age (>50 years). Exposures were investigated separately; only primary exposures are reported (aRR). T1, December 2020/January 2021; T2, March 2021; T3, May/June 2021; T4, Sept/October 2021; T5, December 2021.

Results for period-specific behavioural factors in pupils were inconsistent and usually not statistically significant, except for a higher risk of seroconversion by December 2021 in primary pupils who participated in extracurricular activities in the fall of 2021 (aRR 1.87; 95% CI 1.30; 2.69). In staff, poor self-reported implementation of IPC measures was associated with an increased risk of seropositivity at T1 in primary and secondary school staff (aRR 2.10; 95% CI 0.98 vs. 4.50; 2.27; 1.00; 5.13), but not during later intervals (Figure 5).

### High-risk contacts

During the whole study period, 602 previously seronegative participants reported a high-risk contact with a confirmed COVID-19 case. The risk of seroconversion in these participants was two times higher than the time-depending risk, with seroconversion rates ranging from 13% to 54%, depending on the test period and age of the exposed participant (Supplementary Table S6). The seroconversion rate was up to four times (in general two times) higher when the contact was an adult compared to a minor. During the study, the location of high-risk contacts changed from mostly at home to increasingly at school (Supplementary Figure S6) and for primary school pupils from contact with adults to increasingly with other pupils. When staff reported a high-risk contact at school, it was mostly with another adult and not with a pupil.

## Discussion

From December 2020 to June 2021, we observed in this nationwide SARS-CoV-2 seroprevalence study among pupils and staff in Belgian schools a steady increase in seroprevalence among all age groups. The cumulative seroprevalence was not significantly different between primary and secondary pupils and secondary staff, but primary staff tested significantly more often positive. Follow-up of primary school pupils continued until December 2021, when they reached a cumulative seroprevalence of 60%. More than half of these pupils were infected during the fourth pandemic wave, which was dominated by the delta variant.

Studies from Switzerland and the UK reported a similar high seroprevalence among primary school pupils by the end of 2021, right before the surge of infections due to the omicron wave [16, 17]. In December 2021, a much lower seroprevalence (13.2%) was observed among school pupils in Canada [18], where masking was mandatory for all pupils and staff [19], while masks were as good as not required in the UK, and both Switzerland and Belgium adopted a comparable mixed, age-dependent approach. Masks in primary schools have been shown to reduce case counts [20] and were found to be associated with seroprevalence [21]. In spite of this different masking approach in Belgian primary and secondary schools, we did not observe in our study a difference in seroprevalence among pupils. In June 2021, Ladhani et al. reported a higher seroprevalence among secondary school pupils and staff than reported in our study, but their observation of a comparable seroprevalence in staff and pupils is similar [22]. In our study, the staff from primary schools had a significantly higher seroprevalence by June 2021. A study in Wales [23] also found that the odds of testing positive were higher in staff from primary schools and in staff compared to their pupils.

Vulnerable pupils and those from lower SES schools were more at risk to test positive in our study. Similar findings were reported for schoolchildren belonging to racial and ethnic minorities in Canada [19], Italian children with foreign citizenship [24], and in low SES families in German schools [21]. Crowding and limited options to isolate were formulated as plausible causes, but neither we nor Zinszer et al. observed an increased risk for seropositivity in larger families or in children who shared bedrooms. In staff, we also detected a higher seroprevalence in schools under lower SES.

While the risk in HCWs has been largely studied, the impact on their children is less well known. Nine months after the pandemic onset, we observed an increased risk, albeit not statistically significant, of past infection in children from HCWs, which confirms our findings from October 2020 [3]. This difference disappeared by June 2021, coinciding with high vaccination coverage in HCWs.

There is no evidence that behavioural determinants were associated with seropositivity during the course of the study. While many families travelled during the summer, we did not find an association with seropositivity. It might be that those travelling also restricted contacts outside of their household [25]. Our finding regarding the use of public transport confirms a study in Berlin [21] where no consistent association with the mode of transport to school could be established. This should be placed in context, given that the public transport occupancy was low and mask-wearing was mandatory before and during the study period.

A history of high-risk contact with a confirmed COVID-19 case, at home, or in school is reported to be a major risk factor for seropositivity by our study and others. The added risk of infection after a low-risk contact in school (e.g. short duration, at a proper distance, wearing mask) was overall negligible. In both adults and children, seroconversion occurred more often when the high-risk contact was an adult. Until April 2021, Verberk et al. [26] reported a lower likelihood of transmission in those under 12 years in a household study. We cannot provide secondary attack rates, given the absence of data of the full group at risk, nor do antibodies provide information on infection-relatedness. Nevertheless, high-risk contacts in staff were mostly reported with colleagues. This confirms the findings from a school study in New York [27] where 51% of cases were transmitted among staff.

Comparing our seroprevalence with COVID-19 community surveillance data in the same age groups and at the same time, we estimated a case underreporting ranging from 2.2 to 4.6. Obviously, this depends heavily on the test strategies. In Belgium, testing was restricted during the second wave, but became widely available in the school setting by March 2021. However, our study demonstrates that even widespread testing does not allow to fully capture the magnitude of the pandemic. In addition, changes in infectiousness and virulence of variants are obstacles to estimate the magnitude of bias in surveillance registers.

We confirm that COVID-19 symptoms are not very informative. In addition, a large proportion of seroconversions in our study was asymptomatic. A systematic review [28] estimated that 15%–42% of paediatric infections are asymptomatic. In a prospective school study in Belgium, 46% of children and 31% of adults did not show any symptoms of infection [29]. It is expected that the re-emergence of seasonal infections will diminish the diagnostic performance of symptoms even more.

The nationwide scope and a large geographical and socioeconomic representative sample of pupils and staff are major strengths of our study. Additionally, the use of a non-invasive oral sample ensured a low threshold for participation in a study with multiple data collection points, particularly among children. Saliva antibody tests have not been used extensively despite studies demonstrating their user-friendliness and diagnostic accuracy [30]. Finally, the longitudinal nature of our study allowed to monitor progress of the pandemic in a single sample and to relate behaviour between time points to seroconversion. Limitations of our study are, first, the inevitable selection and response bias. While attrition of individual participants was not related to seropositivity, primary schools with a higher SES were more likely to participate in the study extension. Secondly, staff vaccinated by June 2021 were excluded from the third test period, as well as staff whose vaccination status was unknown. This resulted in a significant reduction in the number of staff. Third, the first samples were collected 9 months after the onset of the pandemic. While it is likely that some (early) infections already seroreverted, most infections in our study population likely occurred during the second wave in fall 2020, immediately before the first test period. Seroreversion during the study period is taken into account by the use of cumulative incidence in the analyses. Fourth, our seroprevalence study measures past infection, but we cannot know when the infections occurred except for a broad time window, nor can we establish a possible direct connection among pupils or between pupils and staff.

Our study monitored the evolution of the pandemic in Belgian schools, providing evidence-based information for public health decisions that could not be studied in a clinical setting. Questions remain regarding COVID-19 in schools and variant changes, interventions, and the role the virus is allowed to play in our society. All these need to be balanced with minimal disruptions to learning opportunities for children.

## Conclusion

Pupils and secondary school staff had a similar increasing seropositivity, but the cumulative incidence was notably higher in primary school staff by June 2021. Determinants mostly relating with seropositivity were a vulnerable SES status in pupils and working in schools within the French-language network, and with a lower SES in staff. About half of seropositive cases reported a previous confirmed infection, while community cases need to be multiplied by three. This confirms the need for community-based seroprevalence studies preferably using minimal invasive test modalities in children. High-risk contacts, especially with adults, doubled the risk of seroconversion.

## Data Availability

Data will be made available for research purposes on reasonable request to the corresponding author (joanna-trees.merckx@mcgill.ca). They are not publicly available because they contain variables such as age, parental occupation, and school, where recognition cannot be excluded.

## Supplementary material

For supplementary material accompanying this paper visit https://doi.org/10.1017/S0950268823000584. click here to view supplementary material

## References

[1] Forbes H, Morton CE, Bacon S, McDonald HI, Minassian C, Brown JP, Rentsch CT, Mathur R, Schultze A, DeVito NJ, MacKenna B, Hulme WJ, Croker R, Walker AJ, Williamson EJ, Bates C, Mehrkar A, Curtis HJ, Evans D, Wing K, Inglesby P, Drysdale H, Wong AYS, Cockburn J, McManus R, Parry J, Hester F, Harper S, Douglas IJ, Smeeth L, Evans SJW, Bhaskaran K, Eggo RM, Goldacre B and Tomlinson LA (2021) Association between living with children and outcomes from covid-19: OpenSAFELY cohort study of 12 million adults in England. British Medical Journal 18(372), n628. 10.1136/bmj.n628 PMC7970340 33737413

[2] Meinck S, Fraillon J and Strietholt R (2022) The Impact of the COVID-19 Pandemic on Education: International Evidence from the Responses to Educational Disruption Survey (REDS). UNESCO Report. Amsterdam: International Association for the Evaluation of Educational Achievement.

[3] Boey L, Roelants M, Merckx J, Hens N, Desombere I, Duysburgh E and Vandermeulen C (2022) Age-dependent seroprevalence of SARS-CoV-2 antibodies in school-aged children from areas with low and high community transmission. European Journal of Pediatrics 181, 571–578. 34455523 10.1007/s00431-021-04222-9 PMC8402965

[4] Government’s Corona Commissariat (Commissariat Corona du Gouvernement/Regeringscommissariaat Corona). “A plan in phases, according to the vaccination strategy” (Un plan par phases, conforme à la stratégie de vaccination). Available at https://health-rack.s3-eu-west-1.amazonaws.com/assets/downloads/faseplanmaart2021FR.pdf (accessed 15 February 2023).

[5] Sciensano Datasets. Available at https://epistat.sciensano.be/covid/ (accessed 15 February 2023).

[6] Kopanja S, Gattinger P, Schmidthaler K, Sieber J, Niepodziana K, Schlederer T, Weseslindtner L, Stiasny K, Götzinger F, Pickl WF, Frischer T, Valenta R and Szépfalusi Z (2022) Characterization of the antibody response to SARS‐CoV‐2 in a mildly affected pediatric population. Pediatric Allergy and Immunology. 33, e13737. 35212039 10.1111/pai.13737 PMC9115525

[7] Serotracker Dashboard. Available at https://www.covid19immunitytaskforce.ca/serotracker/ (accessed 6 July 2022).

[8] Clarke KE, Jones JM, Deng Y, Nycz E, Lee A, Iachan R, Gundlapalli AV, Hall AJ and MacNeil A (2022) Seroprevalence of infection-induced SARS-CoV-2 antibodies – United States, September 2021–February 2022. Morbidity and Mortality Weekly Report. 71, 606–608. 35482574 10.15585/mmwr.mm7117e3 PMC9098232

[9] Leerlingkenmerken Vlaanderen. Available at https://www.vlaanderen.be/statistiek-vlaanderen/onderwijs-en-vorming/leerlingenkenmerken/metadata-leerlingenkenmerkenLeerlingenkenmerken. Moniteur Belge. Available at https://justice.belgium.be/fr/service_public_federal_justice/organisation/moniteur_belge (accessed 3 May 2022).

[10] Sciensano. Community cases. Available at https://epistat.wiv-isp.be/covid/ (accessed 3 May 2022).

[11] Triest D, Geebelen L, De Pauw R, De Craeye S, Vodolazkaia A, Verbrugghe M, Magerman K, Robben LL, Pannus P, Neven K, Ramaekers D, Van Gucht S, Dierick K, Van Loon N, Goossens ME and Desombere I (2021) Performance of five rapid serological tests in mild-diseased subjects using finger prick blood for exposure assessment to SARS-CoV-2. Journal of Clinical Virology 142, 104897. 34304089 10.1016/j.jcv.2021.104897 PMC8282933

[12] Leclercq V, Van den Houte N, Gisle L, Roukaerts I, Barbezange C, Desombere I, Duysburgh E and Van der Heyden J (2022) Prevalence of anti-SARS-CoV-2 antibodies and potential determinants among the Belgian adult population: Baseline results of a prospective cohort study. Viruses 14, 920. 35632663 10.3390/v14050920 PMC9147735

[13] World Health Organization Seroepidemiology Technical Working Group (2021) ROSES‐S: Statement from the World Health Organization on the reporting of seroepidemiologic studies for SARS‐CoV‐2. Influenza and Other Respiratory Viruses 15, 561–568. 34173715 10.1111/irv.12870 PMC8404052

[14] Vandenbroucke JP, Von Elm E, Altman DG, Gøtzsche PC, Mulrow CD, Pocock SJ, Poole C, Schlesselman JJ and Egger M (2007) Strobe initiative. Strengthening the reporting of observational studies in epidemiology (STROBE): Explanation and elaboration. PLoS Medicine 4, e297. 17941715 10.1371/journal.pmed.0040297 PMC2020496

[15] Study Protocol. Available at https://www.sciensano.be/en/biblio/prevalence-and-incidence-antibodies-against-sars-cov-2-children-and-school-staff-measured-one-year (accessed 3 May 2022).

[16] Haile SR, Raineri A, Rueegg S, Radtke T, Ulyte A, Puhan MA and Kriemler S (2023) Heterogeneous evolution of SARS-CoV-2 seroprevalence in school-age children: Results from the school-based cohort study ciao Corona in November–December 2021 in the canton of Zurich. Swiss Medical Weekly 153, 40035. 36787493 10.57187/smw.2023.40035

[17] Office for National Statistics, UK. COVID-19 schools infection survey, England: Pupil antibody data and vaccine sentiment, March to April 2022. Available at https://www.ons.gov.uk/peoplepopulationandcommunity/healthandsocialcare/conditionsanddiseases/bulletins/covid19schoolsinfectionsurveyengland/pupilantibodiesandvaccinesentimentmarch2022 (accessed 14 July 2022).

[18] Data from Study Communication. Available at https://www.covid19immunitytaskforce.ca/encore-study-releases-third-round-of-results-on-sars-cov-2-infection-in-children-in-montreal/ (accessed 14 July 2022).

[19] Zinszer K, McKinnon B, Bourque N, Pierce L, Saucier A, Otis A, Cheriet I, Papenburg J, Hamelin MÈ, Charland K, Carbonneau J, Zahreddine M, Savard A, Fortin G, Apostolatos A, Haley N, Ratté N, Laurin I, Nguyen CT, Conrod P, Boivin G, De Serres G and Quach C (2021) Seroprevalence of SARS-CoV-2 antibodies among children in school and day care in Montreal, Canada. JAMA Network Open 4, e2135975. 34812845 10.1001/jamanetworkopen.2021.35975 PMC8611475

[20] Donovan CV, Rose C, Lewis KN, Vang K, Stanley N, Motley M, Brown CC, Gray FJ Jr, Thompson JW, Amick BC III, Williams ML, Thomas E, Neatherlin J, Zohoori N, Porter A and Cima M (2022) SARS-CoV-2 incidence in K–12 school districts with mask-required versus mask-optional policies—Arkansas, August–October 2021. Morbidity and Mortality Weekly Report 71, 384–389. 35271560 10.15585/mmwr.mm7110e1 PMC8912000

[21] Theuring S, Thielecke M, van Loon W, Hommes F, Hülso C, von der Haar A, Körner J, Schmidt M, Böhringer F, Mall MA, Rosen A, von Kalle C, Kirchberger V, Kurth T, Seybold J, Mockenhaupt FP and BECOSS Study Group (2021) SARS-CoV-2 infection and transmission in school settings during the second COVID-19 wave: A cross-sectional study, Berlin, Germany, November 2020. Eurosurveillance 26, 2100184. 34448448 10.2807/1560-7917.ES.2021.26.34.2100184 PMC8393892

[22] Ladhani SN, Ireland G, Baawuah F, Beckmann J, Okike IO, Ahmad S, Garstang J, Brent AJ, Brent B, Aiano F, Amin-Chowdhury Z, Kall M, Borrow R, Linley E, Zambon M, Poh J, Warrener L, Lackenby A, Ellis J, Amirthalingam G, Brown KE and Ramsay ME (2022) Emergence of the delta variant and risk of SARS-CoV-2 infection in secondary school students and staff: Prospective surveillance in 18 schools, England. EClinicalMedicine 45, 101319. 35233517 10.1016/j.eclinm.2022.101319 PMC8882000

[23] Thompson DA, Abbasizanjani H, Fry R, Marchant E, Griffiths L, Akbari A, Hollinghurst J, North L, Lyons J, Torabi F, Davies G, Gravenor MB and Lyons RA (2021) Staff–pupil SARS-CoV-2 infection pathways in schools in Wales: A population-level linked data approach. British Medial Journal Paediatrics Open 5, e001049. 10.1136/bmjpo-2021-001049 PMC8111870 34192199

[24] Lazzerini M, Benvenuto S, Mariani I, Fedele G, Leone P, Stefanelli P, Vittori G, Schreiber S, Tommasini A, Rezza G, Barbi E and Comar M (2022) Evolution of SARS-CoV-2 IgG seroprevalence in children and factors associated with seroconversion: Results from a multiple time-points study in Friuli-Venezia Giulia Region, Italy. Children 9, 246. 35204966 10.3390/children9020246 PMC8870333

[25] Paulsen M, Scharff AZ, de Cassan K, Sugianto RI, Blume C, Blume H, Christmann M, Hauß C, Illig T, Jonczyk R, Klopp N, Kopfnagel V, Lichtinghagen R, Lucas H, Luhr A, Mutschler F, Pietschmann T, Pott PC, Prokein J, Schaefer P, Stahl F, Stanislawski N, von der Born J, Schmidt BMW, Heiden S, Stiesch M, Memaran N and Melk A (2022) Children and adolescents’ behavioral patterns in response to escalating COVID-19 restriction reveal sex and age differences. Journal of Adolescent Health 70, 378–386. 10.1016/j.jadohealth.2021.11.021 PMC8610846 34972613

[26] Verberk JD, de Hoog ML, Westerhof I, van Goethem S, Lammens C, Ieven G, de Bruin E, Eggink D, Bielicki JA, Coenen S, van Beek J, Bonten MJM, Goossens H and Bruijning-Verhagen PCJL (2022) Transmission of SARS-CoV-2 within households: A remote prospective cohort study in European countries. European Journal of Epidemiology 37, 549–561. 35644003 10.1007/s10654-022-00870-9 PMC9146817

[27] Varma JK, Thamkittikasem J, Whittemore K, Alexander M, Stephens DH, Arslanian K, Bray J and Long TG (2021) COVID-19 infections among students and staff in New York City public schools. Pediatrics 147, e2021050605. 33688033 10.1542/peds.2021-050605

[28] Viner RM, Ward JL, Hudson LD, Ashe M, Patel SV, Hargreaves D and Whittaker E (2021) Systematic review of reviews of symptoms and signs of COVID-19 in children and adolescents. Archives of Disease in Childhood 106, 802–807. 10.1136/archdischild-2020-320972 33334728

[29] Meuris C, Kremer C, Geerinck A, Locquet M, Bruyère O, Defêche J, Meex C, Hayette MP, Duchene L, Dellot P, Azarzar S, Maréchal N, Sauvage AS, Frippiat F, Giot JB, Léonard P, Fombellida K, Moutschen M, Durkin K, Artesi M, Bours V, Faes C, Hens N and Darcis G (2021) Transmission of SARS-CoV-2 after COVID-19 screening and mitigation measures for primary school children attending school in Liège, Belgium. JAMA Network Open 4, e2128757. 34636913 10.1001/jamanetworkopen.2021.28757 PMC8511974

[30] Dobaño C, Alonso S, Fernández de Sevilla M, Vidal M, Jiménez A, Pons Tomas G, Jairoce C, Melé Casas M, Rubio R, Hernández García M, Ruiz-Olalla G, Girona-Alarcón M, Barrios D, Santano R, Mitchell RA, Puyol L, Mayer L, Chi J, Rodrigo Melero N, Carolis C, Garcia-Miquel A, Bonet-Carne E, Claverol J, Cubells M, Fortuny C, Fumadó V, Jou C, Muñoz-Almagro C, Izquierdo L, Bassat Q, Gratacós E, Aguilar R, García-García JJ, Moncunill G and Jordan I (2021) Antibody conversion rates to SARS-CoV-2 in saliva from children attending summer schools in Barcelona, Spain. British Medical Journal Medicine 19, 309. 10.1186/s12916-021-02184-1 PMC8608564 34809617

