# Supplementary material for: SARS-CoV-2 seroprevalence and determinants for salivary seropositivity among pupils and school staff: a prospective cohort study
Source: Epidemiol Infect. 2023 Apr 24;151:e75. doi: 10.1017/S0950268823000584 (PMC10203531; doi:10.1017/S0950268823000584)
Supplement: Supplementary file 1 [file S0950268823000584sup001.docx]

*Epidemiology and Infection*

**SARS-CoV-2 seroprevalence and determinants for salivary seropositivity among pupils and school staff : A prospective cohort study**

Joanna Merckx, Milena Callies, Ines Kabouche, Isabelle Desombere^,^ Els Duysburgh, Mathieu Roelants

**Supplementary Material**

**Supplementary Figure S1: Directed Acyclic Graph (DAG)**

**DAG pupils**

X: Exposures and Confounders

School language network^1^

School level (age group) ^1^

Community exposure^1^

School SES level^1^

Sex^1^

Vulnerable SES^1^

X: Exposures

high-risk contact

(family case, school case, …)

parental education

ease of living/budget

immigrant status

parental occupation

Comorbidity

Family composition

Elderly in family

Family size

Shared bedroom

Y: sero-positivity

X: Time dependent exposures T_1-5_

Extracurricular activities

Public transport used

Travel

Summer camp

^1^variables included in the adjusted model

**DAG staff**

X: Exposures and Confounders

School language network^1^

School level (age group) ^1^

Community exposure^1^

X: Exposures

high-risk contact

(family case, school case, …)

School SES level^1^

Sex^1^

Age^1^

Comorbidity

Staff function

Y: sero-positivity

X: Time dependent exposures T_1-5_

PPE use

Public transport used

Travel

^1^variables included in the adjusted model

## **Supplementary Figure S2: Flow chart of sample selection from all Belgian schools providing general education**

1,703,060 children 6-18 year-old Belgium

5,689 schools

1,562,103 pupils

240,409 staff members

**Elementary schools Secondary schools**

4,241 schools

768,158 pupils

118,695 staff members

1,448 schools

793,945 pupils

121,714 staff members

41 random selected geographic clusters

98 schools contacted 108 schools contacted

44 schools accepted

1,680 pupils invited

1,073 staff members invited

40 schools accepted

4,541 pupils invited

2,355 staff members invited

## **Supplementary Figure S3: Map of Belgium showing the geographical distribution of schools included in the study. Each circle represents a randomly selected district where one primary and one secondary school was recruited. Blue circles represent districts where the primary school only participated in the main study (December 2020 – June 2021).**


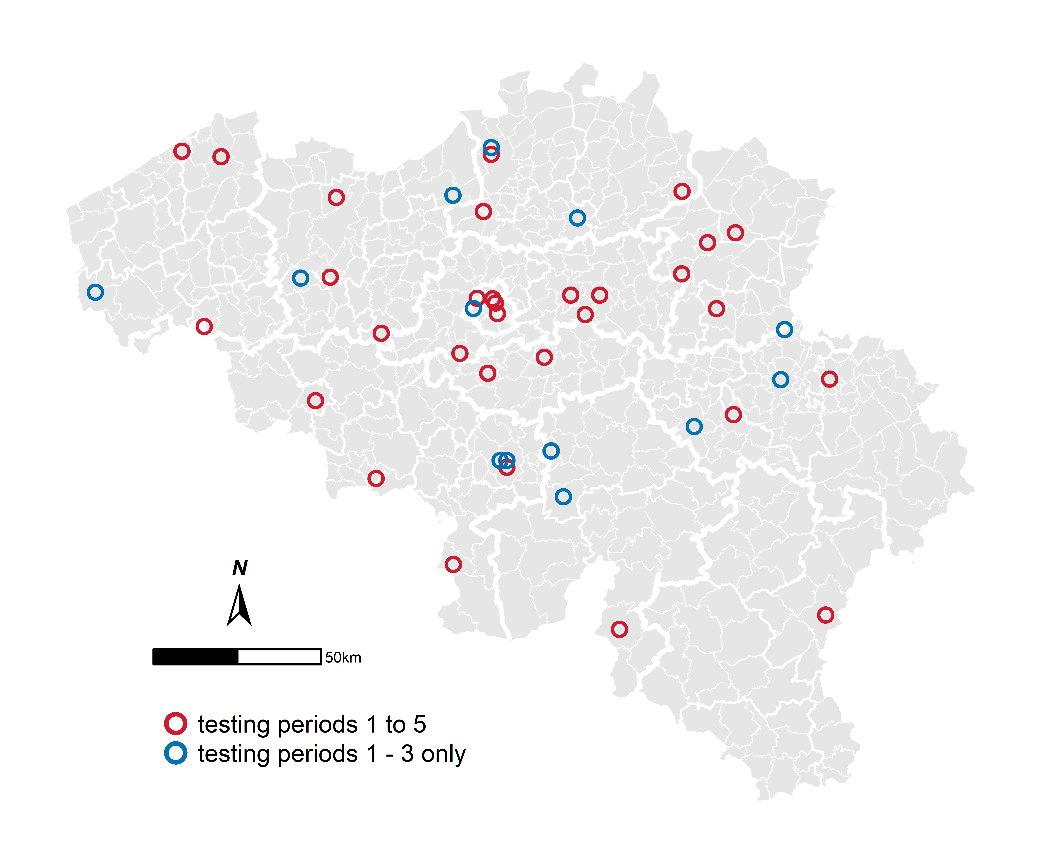


## **Supplementary Table S1: General characteristics (N, %) of primary school pupils included in the main study (December 2020 – June 2021), in the study extension (September – December 2021); and characteristics of primary pupils from school who participated throughout (main study + study extension, from December 2020 – December 2021) and from schools who did not participate to the study extension.**

|  | **Main study** | **Study extension** | **Main + extension** | **Main only (drop out)** |
| --- | --- | --- | --- | --- |
| **Total Number** | 705 | 501 | 321 | 384 |
| Sex (F/M, %F) | 327/378 (46%) | 254/247 (51%) | 161/160 (50%) | 166/218 (43%) |
| Age, median (in years) (range)^1^ | 9 (6-12) | 8 (6-10) | 9 (7-10) | 9 (6-12) |
| Language school board  Dutch/French | 378/327  (54%/46%) | 327/174  (65%/35%) | 195/126  (61%/39%) | 183/201  (48%/52%) |
| School SES  Low  Intermediate  High | 252 (36%)  260 (37%)  193 (27%) | 121 (24%)  198 (40%)  182 (36%) | 79 (25%)  127 (40%)  115 (36%) | 173 (45%)  133 (35%)  78 (20%) |
| Vulnerable SES^2^ | 174 (30%) | 104 (23%) | 69 (23%) | 105 (37%) |

^1^age is missing for 1 participant from the main study and for 5 in the study extension.

^2^ A vulnerable SES is defined as one or more of the following criteria: a lower education of mother or father, unemployed mother or father, household monthly budget <1500EU, financial ease: difficult, language at home does not include Dutch, French or German

## **Supplementary Table S2: Seroprevalence and cumulative seroprevalence by subgroup and test period**

| **Test period** | | **n** | **Seroprevalence (95%CI)** | | **Cumulative seroprevalence (95%CI)** | |
| --- | --- | --- | --- | --- | --- | --- |
| **Pupils, primary** | | | | | | |
| 1 | DEC-JAN | 479 | 11.0 | (7.6 – 15.9) | 11.0 | (7.6 – 15.9) |
| 2 | MAR | 648 | 17.1 | (13.3 – 21.9) | 19.0 | (15.0 – 24.0) |
| 3 | MAY-JUN | 664 | 15.4 | (12.2 – 19.6) | 23.9 | (19.7 – 29.0) |
| 4 | SEP-OCT | 478 | 26.6 | (21.5 – 32.8) | 32.4 | (27.1 – 38.7) |
| 5 | DEC | 432 | 50.9 | (43.7 – 59.2) | 60.4 | (53.4 – 68.3) |
| **Pupils, secondary** | | | | | | |
| 1 | DEC-JAN | 451 | 13.6 | (9.9 – 18.5) | 13.6 | (9.9 – 18.5) |
| 2 | MAR | 515 | 18.0 | (13.6 – 23.8) | 21.6 | (16.8 – 27.7) |
| 3 | MAY-JUN | 525 | 17.2 | (13.1 – 22.7) | 26.4 | (21.3 – 32.7) |
| **Staff, primary** | | | | | | |
| 1 | DEC-JAN | 305 | 16.1 | (12.2 – 21.3) | 16.1 | (12.2 – 21.3) |
| 2 | MAR | 365 | 22.8 | (18.6 – 27.9) | 28.2 | (24.0 – 33.1) |
| 3 | MAY-JUN | 193 | 26.4 | (20.7 – 33.7) | 38.9 | (32.5 – 46.4) |
| **Staff, secondary** | | | | | | |
| 1 | DEC-JAN | 325 | 13.2 | (10.1 – 17.3) | 13.2 | (10.1 – 17.3) |
| 2 | MAR | 321 | 14.6 | (11.5 – 18.6) | 19.0 | (15.7 – 23.0) |
| 3 | MAY-JUN | 175 | 15.9 | (11.6 – 21.8) | 24.2 | (20.3 – 28.8) |

Test periods: DEC-JAN: 2020-12-03 to 2021-01-28; MAR: 2021-03-01 to 2021-03-26; MAY-JUN: 2021-05-17 to 2021-06-11; SEP-OCT: 2021-09-20 to 2021-10-08; DEC: 2021-12-07 to 2021-12-17.

n= number of valid test results by test period and age group.

# **Supplementary Table S3: Risk differences (RD) for past infection (cumulative seroprevalence) in primary school vs. secondary schools and staff vs. pupils point-estimate, corrected for clustering.**

RD (in percentage points) in seroprevalence in secondary vs primary school in pupils and staff and vice versa

|  |  | **Secondary vs Primary** | | **Staff vs Pupils** | |
| --- | --- | --- | --- | --- | --- |
|  | 2021 | Pupils | Staff | Primary | Secondary |
| Test period 1 | DEC-JAN | 2.9 (-3.1; 8.9) | -2.8 (-8.6; 2.9) | 5.3 (0.0; 10.6) | 0.3 (-5.0; 5.7) |
| Test period 2 | MAR | 2.6 (-4.4; 9.6) | -9.2 (-15.0; -3.4) | 8.9 (3.5; 14.3) | -2.2 (-7.8; 3.3) |
| Test period 3 | MAY-JUN | 2.4 (-4.8; 9.7) | -14.5 (-22.7; -6.3) | 14.3 (6.6; 22.1) | -0.4 (-7.8; 7.0) |

Test periods: DEC-JAN: 2020-12-03 to 2021-01-28; MAR: 2021-03-01 to 2021-03-26; MAY-JUN: 2021-05-17 to 2021-06-11; SEP-OCT: 2021-09-20 to 2021-10-08; DEC: 2021-12-07 to 2021-12-17.

**Supplementary Figure S4: Proportion of primary and secondary school pupils and staff who reported COVID-19 related symptoms in the period before testing, in those who tested newly positive (pos; sensitivity) versus those who remained negative (neg).**


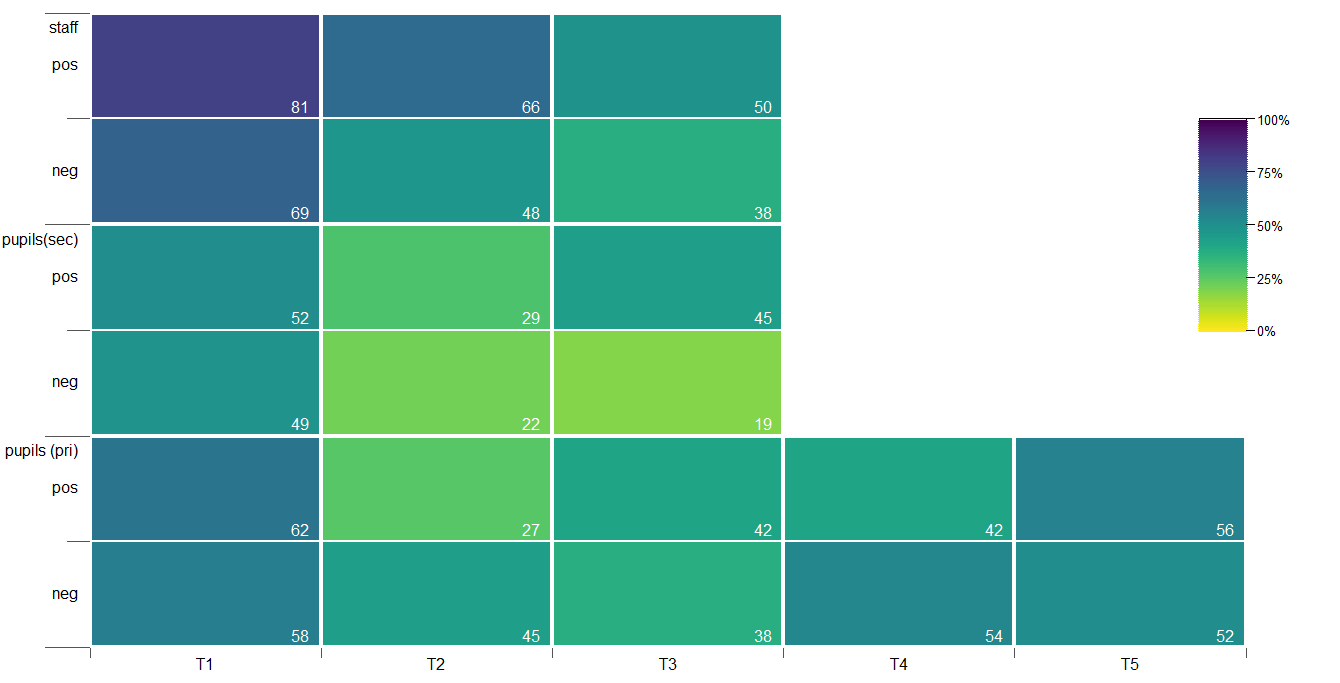


Heat map presenting the proportion of initially seronegative participants who reported any symptom potentially related to COVID-19 (i.e.. fever, cough, shortness of breath, chest pain, headache, nasal congestion or runny nose, throat pain, earache, myalgia, fatigue, abdominal pain, vomit, diarrhoea, nausea, rash, red or sore eyes, change or loss of smell or taste), in the period immediately before each of the 5 test periods (X-axis). Only participants who did not test positive before were included in this analysis. On the Y-axis participants are categorized by age group and by test result (**pos** = presence of RBD antibodies, indicative for the sensitivity of symptoms; **neg** = no RBD antibodies detected, indicative for 1 – sensitivity).

T1: December 2020/January 2021; T2: March 2021; T3: May/June 2021; T4: Sept/October 2021; T5: December 2021.

## **Supplementary Table S4: Socio-demographic determinants of seropositivity at baseline**

1. Pupils

|  | **Number** | | **Seroprevalence (95%CI)** | **RR (95%CI)** | **aRR (95%CI)** |
| --- | --- | --- | --- | --- | --- |
|  | **Total** | **Tested** |  |  |  |
| All pupils | 1274 | 930 | 12.4 (9.7-15.8) | - | - |
| School type: Primary | 713 | 479 | 11.0 (7.6-15.9) | ref | ref |
| Secondary | 576 | 451 | 13.6 (9.9-18.5) | 1.09 (0.67-1.78) | 1.17 (0.73-1.90) |
| Language network: French | 566 | 370 | 16.6 (11.8-23.5) | ref | ref |
| Dutch | 723 | 560 | 9.2 (6.9-12.3) | 0.57 (0.36-0.91) | 0.71 (0.19-2.63) |
| Community exposure (district) | 1289 | 930 | NA | 1.15 (1.05-1.25) | 1.04 (0.78-1.39) |
| School SES tertile: lowest | 421 | 318 | 16.4 (11.0-24.2) | ref | ref |
| middle | 445 | 305 | 11.4 (7.6-17.1) | 0.92 (0.53-1.60) | 1.06 (0.60-1.89) |
| highest | 423 | 307 | 8.8 (6.1-12.7) | 0.59 (0.32-1.07) | 0.64 (0.34-1.17) |
| Sex: male | 679 | 510 | 13.0 (9.6-17.4) | ref | ref |
| female | 610 | 420 | 11.4 (8.8-14.8) | 0.93 (0.67-1.30) | 0.92 (0.65-1.32) |
| Vulnerable SES: no | 759 | 567 | 10.3 (7.6-14.0) | ref | ref |
| yes | 296 | 209 | 16.0 (12.0-21.4) | 1.58 (1.09-2.30) | 1.47 (0.98-2.19) |
| Maternal education: low | 48 | 33 | 18.0 (8.9-36.3) | ref | ref |
| secondary | 299 | 212 | 13.2 (9.3-18.7) | 0.72 (0.32-1.61) | 0.86 (0.37-2.00) |
| higher | 669 | 505 | 10.9 (7.8-15.1) | 0.60 (0.28-1.30) | 0.80 (0.35-1.82) |
| Maternal employment: no | 110 | 78 | 14.3 (8.1-25.3) | Ref | ref |
| yes | 856 | 641 | 11.4 (8.6-15.2) | 0.79 (0.44-1.43) | 1.32 (0.64-2.71) |
| Family budget: low | 239 | 170 | 10.6 (7.1-15.9) | ref | ref |
| high | 518 | 387 | 11.0 (7.8-15.7) | 1.06 (0.65-1.73) | 1.64 (0.94-2.87) |
| Origin: Belgium | 756 | 560 | 10.4 (7.6-14.1) | ref | ref |
| EU | 173 | 123 | 13.6 (8.4-21.8) | 1.34 (0.77-2.34) | 1.28 (0.74-2.22) |
| non-European | 119 | 88 | 18.0 (11.0-29.6) | 1.73 (0.97-3.10) | 1.48 (0.82-2.66) |
| Shared bedroom: no | 726 | 537 | 10.8 (7.9-14.7) | ref | ref |
| yes | 303 | 223 | 13.6 (9.8-18.8) | 1.23 (0.86-1.76) | 1.10 (0.72-1.68) |
| Family type: other | 257 | 194 | 11.5 (7.5-17.6) | ref | ref |
| core family | 772 | 566 | 12.1 (9.2-16.1) | 1.09 (0.68-1.75) | 1.35 (0.80-2.27) |
| Family size: < 6 | 880 | 649 | 12.2 (9.2-16.1) | ref | ref |
| > 5 | 146 | 108 | 9.6 (4.9-18.7) | 0.82 (0.39-1.71) | 0.68 (0.32-1.45) |
| Family member >65y: no | 1265 | 908 | 12.4 (9.7-15.9) | ref | ref |
| yes | 24 | 22 | 16.5 (7.2-38.1) | 1.15 (0.42-3.11) | 1.12 (0.39-3.17) |
| Comorbidity: no | 992 | 727 | 11.3 (8.6-14.9) | ref | ref |
| yes | 50 | 41 | 19.4 (11.0-34.1) | 1.83 (0.94-3.57) | 1.59 (0.77-3.29) |
| Occupational risk (parent): low | 405 | 301 | 8.2 (5.7-11.8) | ref | ref |
| high | 446 | 335 | 12.6 (9.2-17.2) | 1.45 (0.97-2.18) | 1.42 (0.94-2.15) |
| HCW parent: no | 695 | 520 | 9.9 (7.4-13.2) | ref | ref |
| yes | 203 | 151 | 14.0 (9.3-21.2) | 1.44 (0.94-2.21) | 1.46 (0.93-2.30) |
| Teaching parent: no | 775 | 581 | 10.9 (8.2-14.5) | ref | ref |
| yes | 123 | 90 | 9.7 (5.4-17.5) | 0.92 (0.48-1.75) | 1.03 (0.56-1.90) |

Total = distribution in all participants with data available; tested = distribution in participants who were tested at baseline. Seroprevalence, RR and aRR determined at baseline. RR: relative risk, aRR: partially adjusted relative risk (adjusted for school type, language network, community exposure, SES tertile, sex and vulnerable SES).

SES: socio-economic status; HCW: health care worker

1. Staff

|  | **Number** | | **Seroprevalence (95%CI)** | **RR (95%CI)** | **aRR (95%CI)** |
| --- | --- | --- | --- | --- | --- |
|  | **Total** | **Tested** |  |  |  |
| All staff | 799 | 630 | 14.6 (12.0-17.8) |  |  |
| School type: Primary | 434 | 305 | 16.1 (12.2-21.3) | ref | ref |
| Secondary | 391 | 325 | 13.2 (10.1-17.3) | 0.82 (0.56-1.21) | 0.84 (0.57-1.24) |
| Language network: French | 383 | 272 | 16.7 (13.1-21.3) | ref | ref |
| Dutch | 442 | 358 | 12.8 (9.4-17.4) | 0.75 (0.51-1.11) | 0.51 (0.23-1.14) |
| Community exposure (district) | 825 | 630 | NA | 1.04 (0.95-1.15) | 0.90 (0.73-1.10) |
| Schools SES: lowest | 279 | 226 | 15.9 (11.9-21.3) | ref | ref |
| middle | 303 | 214 | 14.0 (10.1-19.3) | 0.88 (0.57-1.36) | 0.89 (0.56-1.42) |
| highest | 243 | 190 | 13.7 (9.0-20.8) | 0.86 (0.51-1.43) | 0.87 (0.53-1.44) |
| Sex: male | 214 | 177 | 12.7 (8.5-19.1) | ref | ref |
| female | 611 | 453 | 15.4 (12.4-19.2) | 1.23 (0.78-1.93) | 1.15 (0.71-1.85) |
| Age | 825 | 630 | NA | 0.99 (0.97-1.01) | 0.99 (0.97-1.01) |
| Staff function: non-teaching | 148 | 117 | 9.9 (6.0-16.4) | ref | ref |
| teaching | 636 | 484 | 15.7 (12.7-19.3) | 1.52 (0.90-2.56) | 1.50 (0.90-2.51) |
| Comorbidity: no | 669 | 510 | 14.9 (12.2-18.2) | ref | ref |
| yes | 96 | 78 | 9.9 (4.5-21.5) | 0.60 (0.26-1.39) | 0.59 (0.25-1.38) |

Total = distribution in all participants with data available; tested = distribution in participants who were tested at baseline. Seroprevalence, RR and aRR determined at baseline. RR: relative risk, aRR: partially adjusted relative risk (adjusted for school type, language network, community exposure, SES tertile, age and sex).

SES: socio-economic status.

## **Supplementary Figure S5: Seropositivity according to socio-demographic characteristics in staff at T3 (May-June 2021)**


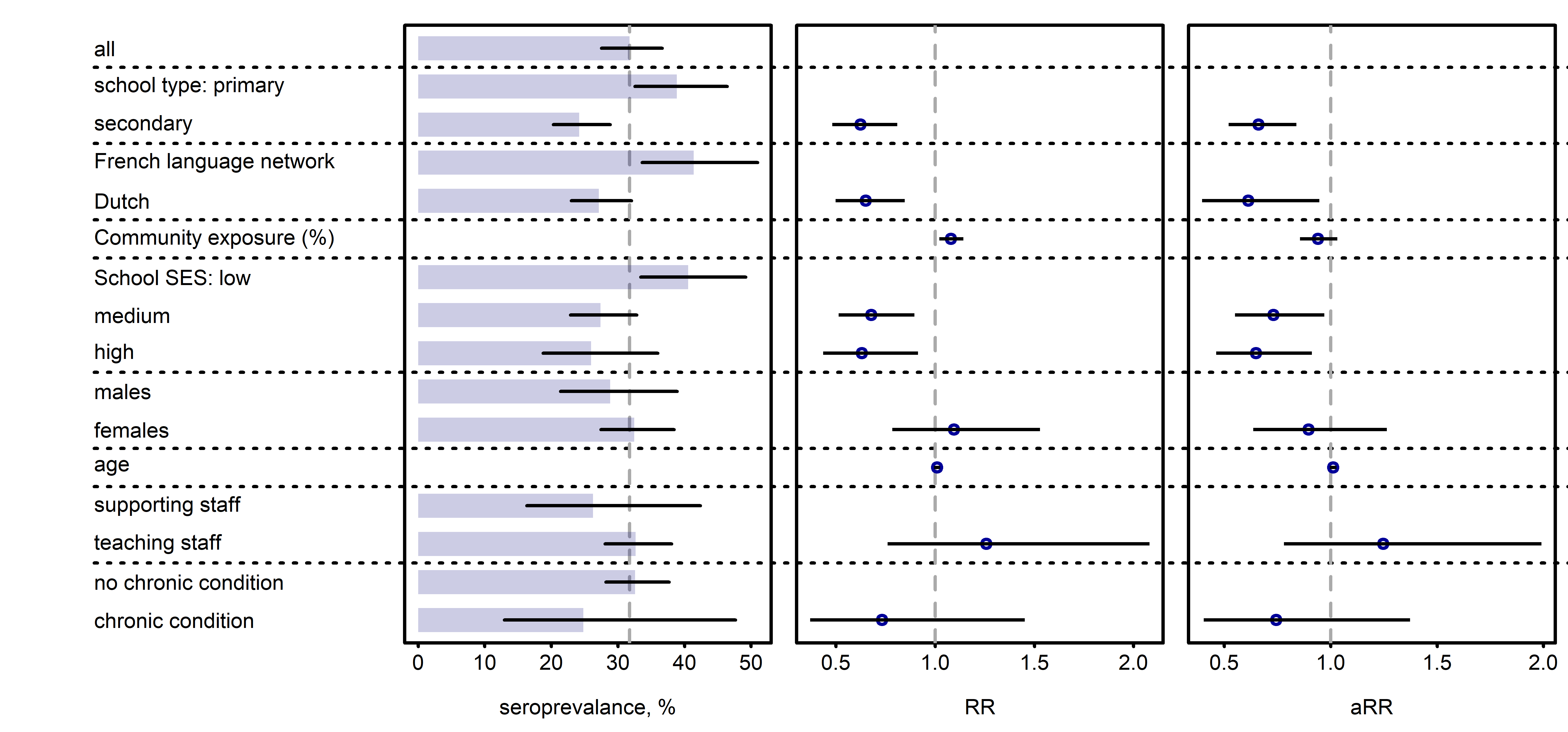


aRR: partially adjusted relative risk (adjusted for school type, language network, community exposure, SES tertile, age and sex).

SES: socio-economic status.

## **Supplementary Table S5: Seropositivity according to socio-demographic characteristics in staff at T3 (May-June 2021)**

|  | Number  Total | Tested | Seroprevalence (95%CI) | RR (95%CI) | aRR (95%CI) |
| --- | --- | --- | --- | --- | --- |
| All staff | 799 | 368 | 31.8 (27.5-36.7) |  |  |
| School type: Primary | 434 | 193 | 38.9 (32.5-46.4) | ref | ref |
| Secondary | 391 | 175 | 24.2(20.3-28.8) | 0.62 (0.48-0.81) | 0.66 (0.52- 0.84) |
| Language network: French | 383 | 121 | 41.4 (33.6-51.0) | ref | ref |
| Dutch | 442 | 247 | 27.1 (23.0-32.0) | 0.65 (0.50-0.85) | 0.61 (0.40- 0.97) |
| Community exposure (district) | 825 | 368 | NA | 1.08 (1.02-1.14) | 0.94 (0.86-1.03) |
| Schools SES: lowest | 279 | 131 | 40.6 (33.4-49.2) | ref | ref |
| middle | 303 | 137 | 27.4 (22.8-32.8) | 0.68 (0.51-0.90) | 0.73 (0.55-0.97) |
| highest | 243 | 100 | 26.0 (18.7-36.0) | 0.63 (0.44-0.91) | 0.65 (0.46-0.91) |
| Sex: male | 214 | 94 | 28.8 (21.4-38.9) | ref | ref |
| female | 611 | 274 | 32.5 (27.4-38.4) | 1.09 (0.78-1.53) | 0.90 (0.64- 1.26) |
| Age | 825 | 368 | NA | 1.01 (0.99-1.03) | 1.01 (1.00-1.03) |
| Staff function: non-teaching | 148 | 50 | 26.3 (16.3-42.4) | ref | ref |
| teaching | 636 | 315 | 32.7 (28.1-38.0) | 1.26 (0.71-2.08) | 1.25 (0.78-1.99) |
| Comorbidity: no | 669 | 331 | 32.6 (28.2-37.7) | ref | ref |
| yes | 96 | 29 | 24.8 (12.9-47.6) | 0.73 (0.37- 1.45) | 0.74 (0.40-1.37) |

Total = distribution in all participants with data available; tested = distribution in participants who were tested at baseline. Seroprevalence, RR and aRR determined at baseline. RR: relative risk, aRR: partially adjusted relative risk (adjusted for school type, language network, community exposure, SES tertile, age and sex).

SES: socio-economic status.

## **Supplementary Table S6: Seroconversion according to type of contact at each test period in susceptible pupils and staff (not previously seropositive): n positieve / n susceptible (percentage)]**

|  | **T1** | **T2** | **T3** | **T4** | **T5** |
| --- | --- | --- | --- | --- | --- |
| **Pupils, primary** | | | | | |
| all | 53/479 (11.1) | 77/602 (12.8) | 38/543 (7.0) | 84/410 (20.5) | 121/295 (41.0) |
| High-risk^1^ contact | 17/56 (30.4) | 9/39 (23.1) | 9/41 (22.0) | 27/78 (34.6) | 54/100 (54.0) |
| Contact at home^2^ | 13/41 (31.7) | 6/17 (35.3) | 7/14 (50.0) | 15/31 (48.4) | 22/39 (56.4) |
| Contact in school^2^ | 2/8 (25.0) | 1/10 (10.0) | 2/15 (13.3) | 9/26 (34.6) | 28/51 (54.9) |
| Contact with minor | 1/7 (14.3) | 3/15 (20.0) | 2/22 (9.1) | 9/37 (24.3) | 35/66 (53.0) |
| Contact with adult | 16/49 (32.7) | 6/24 (25.0) | 7/19 (36.8) | 18/41 (43.9) | 19/34 (55.9) |
| **Pupils, secondary** | | | | | |
| all | 58/451 (12.9) | 52/460 (11.3) | 31/422 (7.3) |  | |
| High-risk^1^ contact | 15/57 (26.3) | 4/22 (18.2) | 4/22 (18.2) |  | |
| Contact at home^2^ | 11/34 (32.4) | 1/12 (8.3) | 3/10 (30.0) |  | |
| Contact in school^2^ | 3/13 (23.1) | 1/5 (20.0) | 0/9 (0.0) |  | |
| Contact with minor | 3/21 (14.3) | 0/8 (0.0) | 2/11 (18.2) |  | |
| Contact with adult | 12/36 (33.3) | 4/14 (28.6) | 2/11 (18.2) |  | |
| **Staff, primary** | | | | | |
| all | 49/305 (16.1) | 57/319 (17.9) | 15/133 (11.3) |  | |
| High-risk^1^ contact | 10/50 (20.0) | 3/23 (13.0) | 5/14 (35.7) |  | |
| Contact at home^2^ | 6/31 (19.4) | 3/10 (30.0) | 2/5 (40.0) |  | |
| Contact in school^2^ | 2/15 (13.3) | 0/12 (0.0) | 2/6 (33.3) |  | |
| Contact with minor | 0/7 (0.0) | 1/9 (11.1) | 1/4 (25.0) |  | |
| Contact with adult | 10/43 (23.3) | 2/14 (14.3) | 4/10 (40.0) |  | |
| **Staff, secondary** | | | | | |
| all | 43/325 (13.2) | 28/288 (9.7) | 12/145 (8.3) |  | |
| High-risk^1^ contact | 13/63 (20.6) | 6/27 (22.2) | 0/10 (0.0) |  | |
| Contact at home^2^ | 7/30 (23.3) | 4/17 (23.5) | 0/4 (0.0) |  | |
| Contact in school^2^ | 2/20 (10.0) | 1/5 (20.0) | 0/3 (0.0) |  | |
| Contact with minor | 0/3 (0.0) | 0/3 (0.0) | 0/2 (0.0) |  | |
| Contact with adult | 13/60 (21.7) | 6/24 (25.0) | 0/8 (0.0) |  | |

^1^High-risk contact: contact with a confirmed case without mask for at least 15 min. at a distance less than 2 m. All confirmed contacts at home were deemed high risk.

^2^numbers by location do not add up because some contacts did not occur at home or in school

T1: December 2020/January 2021; T2: March 2021; T3: May/June 2021; T4: Sept/October 2021; T5: December 2021.

## **Supplementary Figure S6: Overview of confirmed contact by location and test period**
